# Reply to Lowen et al., “The value of virological research in wildlife”

**DOI:** 10.1128/mbio.03756-25

**Published:** 2026-02-18

**Authors:** Marc Lipsitch, Aishani V. Aatresh

**Affiliations:** 1 Department of Epidemiology, Center for Communicable Disease Dynamics, Harvard TH Chan School of Public Health 1857 , Boston, Massachusetts, USA; Tsinghua University, Beijing, China

## REPLY

In the United States, public health initiatives have long relied on public funding and produced science and technologies to underwrite and implement public policies. Identifying priorities for such research and associated efforts to produce benefits for society, broadly construed, is an important activity best conducted not only by the funders of and recipients of funding, but by broader communities of experts and the public (1, 2). We agree with Drs. Lowen, Runstadler, and Lakdawala (3) that “simple cataloging” of “potential zoonotic viruses” “likely has little direct public health benefit.” Nonetheless, hundreds of millions of dollars were spent and billions more proposed with this objective (4), making evaluation a pressing need. We also agree with their statement that “the expectation that licensed vaccines will follow from virus discovery is unrealistic” (3). However, vaccine development has been cited explicitly by key proponents of such work as one of its benefits (5–7). It seems only fair to take defenders of this position at their word and evaluate their claims rigorously, as our paper does. Still, in doing so, “[w]e do not equate the lack of direct MCM development with a lack of value for virus prospecting” (8).

The critics’ next concern is that “the arguments made overlook the profound benefits of fundamental research” (3). The benefits of fundamental research, which we acknowledge, argue in favor of undertaking fundamental research in general, not viral prospecting in particular. In fact, viral prospecting is not in the classic sense “fundamental research,” but is rather applied research in the form of natural history with modern tools, directed at a very particular end. Initiatives in its name are part of a longer history of large scientific and technological projects designed to address public problems—in this case, threats of infectious diseases. Viral prospecting is predicated on the notions that scientists can reasonably anticipate what benefits will come from this effort, and that these benefits justify such research for public health policy. In this regard, viral prospecting should not be evaluated as curiosity-driven basic research with high potential to generate truly unanticipated benefits. Discussions of which research holds high priority should focus on its anticipated, specific benefits to protect societies, as well as the consequences of falling short. Our article is a rigorous empirical analysis of how and to what extent viral prospecting has lived up to the expectations attached to it—to produce knowledge that demonstrably protects people against risks of infectious diseases, especially on a global scale—and where adjustments might be beneficial for science and society alike.

While it is impossible to guard against misunderstanding of any written document, we are pleased to reemphasize—as the critics invite us to do with their final concern—that our analysis applies to viral prospecting, the activity of cataloging novel viruses in wildlife populations. We specify that our article “focuses on viral prospecting to identify novel threats rather than zoonotic surveillance as a broader category of efforts to characterize viral diversity, build technical capacity, or inform behavioral interventions and basic research.” This specific activity has received substantial investment, as noted, and there have been calls to increase that investment by an order of magnitude (6). It has recently lost support in several quarters due to concerns about risk and cost, as outlined in our article and citations therein. In this vein, our article speaks to the value of “attending to under-considered existing phenomena (e.g., endemic viruses, public health infrastructure, and socioeconomic disparities)” (8). The text notably acknowledges the value of such efforts to inform and motivate prevention, including ongoing studies in animals for viruses of known concern, such as influenza in birds and Nipah virus in bats.

In conclusion, we agree with Drs. Lowen, Runstadler, and Lakdawala that viral threats fundamentally involve interlinkages among humans, animals, and the environment. However, our article underscores questions about how we should account for such interlinkages, both in the form of the questions we ask about them and the interventions we design to address them. In contrast to our critics’ statement that “[r]esearch is our best defense against pandemic threats” (3), we consider research to be but one essential part of preparedness. It exists alongside efforts to strengthen health systems, improve governance structures, remedy socioeconomic conditions that drive spillovers, and ensure wider availability of countermeasures. Without these pillars, research will fall short of its potential to enable the public health advances we all seek.
